# Clinical Effect of Intravenous Vitamin C on Viral Myocarditis in Children: A Systematic Review and Meta-Analysis

**DOI:** 10.1155/2019/3082437

**Published:** 2019-04-23

**Authors:** Shuangdi Chen, Wenli Zhao, Binjie Zhang, Yijun Jia, Shihua Wu, Botao Zhong, Xuerong Yu, Xueying Wang, Yu Hao, Hongwu Wang, Ye Zhao, Kaito Mizuno, Huaien Bu, Yiider Tseng

**Affiliations:** ^1^College of Traditional Chinese Medicine, Tianjin University of Traditional Chinese Medicine, Tianjin 300193, China; ^2^Department of Neurology, Nankai Hospital, Tianjin Academy of Integrative Medicine, Tianjin 300100, China; ^3^Graduate School, Tianjin University of Traditional Chinese Medicine, Tianjin 300193, China; ^4^Department of Library, Tianjin University of Traditional Chinese Medicine, Tianjin 300193, China; ^5^Department of Chemical Engineering, University of Florida, Gainesville, Florida 32611, USA; ^6^Faculty of Health Science, Suzuka University of Medical Science, Suzuka 510-0293, Japan

## Abstract

**Objective:**

To comprehensively compare the effects of conventional therapy combined with intravenous vitamin C and conventional therapy on viral myocarditis in children through a meta-analysis.

**Methods:**

Relevant articles including clinical trials of normal treatment combined with intravenous vitamin C and conventional therapy for viral myocarditis in children that were published between January 2000 and February 2018 were selected from PubMed, Cochrane Library, China National Knowledge Infrastructure, China Science and Technology Journal Database, and WANFANG database. The quality of the included studies was assessed using the Cochrane systematic review method (version 5.1.0); data quality was evaluated by two independent researchers. The total effective rate; LDH, CK, and CK-MB levels; and other indicators were analyzed using Rev Man 5.3 software.

**Results:**

Eight studies were eligible for this meta-analysis, which included a total of 426 patients in the treatment group and 363 patients in the control group. The meta-analysis results of six studies showed that the total effective rate of intravenous vitamin C combined with conventional therapy was higher than that of conventional therapy alone [Z = 5.46, 95% confidence interval (CI): 1.21 (1.13 to 1.30),* P* < 0.00001]; that of five studies showed that LDH levels were lower in children receiving intravenous vitamin C combined with conventional therapy than in those receiving conventional therapy alone [Z = 3.70, 95% CI: −1.88 (−2.88 to −0.88),* P* = 0.0002]; that of three studies showed that CK levels were lower in children receiving intravenous vitamin C combined with conventional therapy than in those receiving conventional therapy alone [Z = 4.21, 95% CI: −0.55 (−0.81 to −0.30),* P* < 0.0001]; that of four studies showed that CK-MB levels were lower in children receiving intravenous vitamin C combined with conventional therapy than in those receiving conventional therapy alone [Z = 13.64, 95% CI: −1.44 (−1.65 to −1.24),* P* < 0.00001]; that of two studies showed that CD3 levels were higher in children receiving intravenous vitamin C combined with conventional therapy than in those receiving conventional therapy alone [Z = 2.45, 95% CI: 0.41 (0.08–0.73),* P* = 0.01]; that of two studies showed no significant difference in changes in CD4 levels between children receiving intravenous vitamin C combined with conventional therapy and those receiving conventional therapy alone [Z = 0.28, 95% CI: −0.21 (−1.69 to 1.28),* P* = 0.78]; and that of two studies showed no significant difference in changes in CD4/CD8 between children receiving intravenous vitamin C combined with conventional therapy and those receiving conventional therapy alone [Z = 0.07, 95% CI: −0.03 (−0.73 to 0.67),* P* = 0.94].

**Conclusion:**

The meta-analysis results showed that intravenous vitamin C combined with conventional therapy is better than the simple, conventional therapy for the treatment of viral myocarditis in children in terms of the total effective rate and LDH, CK, and CK-MB levels.

## 1. Introduction

Viral myocarditis is one of the most common pediatric heart diseases. An outbreak of viral myocarditis with unknown origin occurred in the village of Da Jing, Yunnan, China, and resulted in four deaths and 37 cases of clustered sudden death, causing widespread concern [1]. Currently, the number of viral myocarditis cases in children is increasing annually; however, the mechanism underlying this disease has not yet been elucidated, and the diagnostic criteria are constantly changing [2, 3]. The clinical manifestations of viral myocarditis mainly include moderate and severe palpitations, chest distress, chest pain or discomfort in the precordial area, shortness of breath, gasp or sighing, fatigue, sweating, diarrhea, lack of energy, pale complexion, and other symptoms [4]. In 1991, China was the first country to use the polymerase chain reaction technology for the detection of viral nucleic acid, which is of great value for early diagnosis and prognosis. Although this disease is caused by a viral infection and can occur in any age group, it occurs more commonly in children aged 3–10 years. The diagnosis is typically based on the characteristics of the cause, clinical symptoms, and signs associated with the heart, laboratory examination of the electrocardiogram, elevated levels of myocardial necrosis markers, abnormal ultrasound and electrocardiogram, and exclusion of other heart diseases. Under normal circumstances, gastrointestinal discomfort or central nervous system symptoms in children should be first considered as pediatric myocarditis [5]. When diagnosed or treated as soon as possible, most children show considerable improvement following treatment, but untimely treatment or improper rehabilitation following illness would lead to a protracted course of disease in some children. However, the diagnosis and treatment of myocarditis require further exploration because there is a lack of substantial relevant clinical research data.

The existing treatment methods are as follows: general treatments, symptomatic treatments, and drug treatments.

According to the latest research, an intravenous injection of vitamin C at a dose based on the conventional therapy may effectively enhance the therapeutic effect of treatment. It is probable that vitamin C can shorten the treatment course of viral myocarditis in children. Moreover, it is possible that this addition of vitamin C can satisfactorily improve clinical symptoms and the electrocardiogram findings and myocardial enzymes [6, 7]. Furthermore, a study has shown that vitamin C is a highly effective and practical nutrient component. The product itself exhibits no toxic side effects and does not cause any mental or psychological stress on the patients, particularly children. Vitamin C also exerts antimicrobial and antiviral effects. [2]. The intravenous injection of vitamin C may increase myocardial contractility, improve left ventricular function, and promote myocardial function recovery in children with myocarditis [8–10]; however, some controversies remain regarding whether the therapeutic effect of vitamin C plus conventional therapy is better than that of conventional therapy at home and abroad. Therefore, we performed an inductive retrieval of related papers and analyzed the effect of vitamin C plus conventional therapy in a meta-analysis.

## 2. Methods

### 2.1. Research Strategy

Relevant articles published between January 2000 and February 2018 were selected from the PubMed, Cochrane Library, China National Knowledge Infrastructure (CNKI), China Science and Technology Journal Database (VIP), and WANFANG database. The keywords were vitamin C and viral myocarditis in children. The search was performed by combining the search terms with the subject words.

### 2.2. Literature Inclusion Criteria

#### 2.2.1. Research Design

Randomized controlled clinical trials were selected regardless of language and publication restrictions.

#### 2.2.2. Research Subjects

Children (age and sex) were diagnosed according to the latest diagnostic criteria for pediatric viral myocarditis [3] published by the World Health Organization.

#### 2.2.3. Intervention

The control group received conventional therapy only, whereas the experimental group received a certain dose of vitamin C intravenously based on the conventional therapy.

#### 2.2.4. Outcome Indicators

Outcome indicators were total effective rate; lactate dehydrogenase (LDH), creatine kinase (CK), creatine kinase isoenzyme (CK-MB), CD3, and CD4 levels; and CD4/CD8 value.

LDH, CK, and CK-MB levels were determined using colorimetric assay, immunosuppression, and enzyme-linked immunosorbent assay, respectively. When LDH level increases, it exhibits tissue specificity in human body parts, indicating that myocardial lesions occur. In humans, CK level shows a rapid increase in cases of myocardial ischemia or subendocardial infarction, and it is thus an important indicator for clinical diagnosis of myocarditis. A significantly elevated serum CK-MB level suggests that the myocardium is significantly affected, and it is typically considered that serum CK-MB level of >6% of total activity is a specific indicator of myocardial injury.

Fasting peripheral venous blood (5 mL) was collected before and after treatment, and serum T cell subsets (CD3+, CD4+, and CD4+/CD8+) levels were detected using flow cytometry. T lymphocytes are effector cells of cellular immunity as well as important immunoregulatory cells, in addition to being a heterogeneous multifunctional population. CD3+ represents the total number of mature T cells in the peripheral blood, and an increased level indicates that T cell immune function is enhanced; CD4+ is a subset of T lymphocyte, which is helpful to induce. The increase of CD4+ indicates the increase of immunoglobulin produced by B lymphocyte and the enhancement of cellular immunity. CD8+ is an inhibitory cell, and its increase indicates immunosuppression.

### 2.3. Document Exclusion Criteria

Exclusion criteria were as follows: (1) republished studies, (2) animal experiment studies, (3) literature review and observational studies, and (4) literature that does not meet the requirements of this study.

### 2.4. Quality Evaluation and Data Extraction

The methodological quality of the included studies was evaluated based on the quality assessment criteria recommended in the Cochrane systematic review manual [11]. The main evaluation criteria included the following: (1) a randomly assigned method, (2) allocation concealment, (3) use of blinding, (4) data integrity, (5) selectively reported results, and (6) the presence of bias. “Low risk” indicates a low risk of bias, “high risk” indicates a high risk of bias, and “unclear risk” indicates that the literature does not provide sufficient information for bias assessment. The data quality was evaluated by two independent researchers. Inconsistent opinions were resolved via a discussion or by soliciting the advice of a third party regarding the inclusion of a particular study.

### 2.5. Statistical Analysis

Data were analyzed using Rev Man 5.3 software. Binary data were assessed using relative risk (RR) to determine the effect amount; continuous data were analyzed using the standardized mean difference (SMD) to determine the effect amount, and both 95% confidence intervals (CIs) were displayed. The P value of the heterogeneity test result displayed in the forest map was used to determine whether the included study was heterogeneous; heterogeneity was assessed using the I^2^ statistic. If the heterogeneity was not significant (*P* ≥ 0.1, I^2^ ≤ 50%), the effect size was combined using a fixed effect model; if the heterogeneity was significant (*P* < 0.1, I^2^ > 50%), the random effects model was used to combine the effect sizes.

## 3. Results

### 3.1. Literature Screening

**Figure 1:**
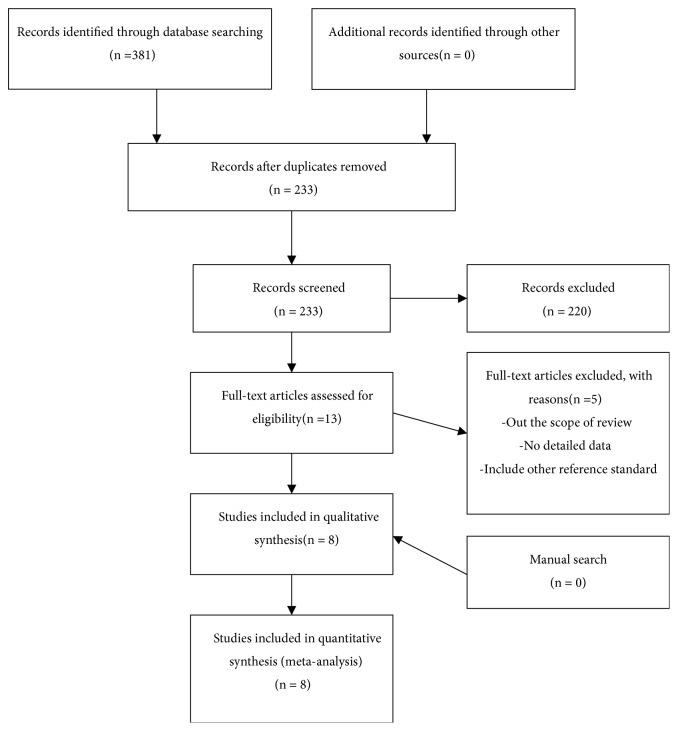
Literature screening flow chart.

Overall, 381 articles were identified, including 357 in Chinese and 24 in English. After excluding duplicated and cross-extracted literature, the titles and abstracts were reviewed, and an additional 347 studies were excluded. Initially, 13 Chinese documents were included (no English studies). After reading the full text, further screening was performed according to the inclusion and exclusion criteria; finally, eight Chinese studies were included. The literature screening process is shown in Figure 1.

### 3.2. Literature Quality Evaluation

The literature information [6–8, 12–16] is presented in Table 1, and the literature quality evaluation is presented in Table 2.

### 3.3. Meta-Analysis Results

#### 3.3.1. Total Effective Rate

**Figure 2:**
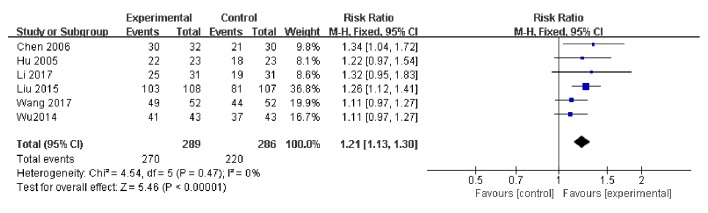
Changes in total effective rate of vitamin C combined with conventional therapy compared with simple treatment.

Overall, six studies used the total effective rate as an indicator of the effectiveness of vitamin C-guided interventions. A total of 490 children with myocarditis were included in this assessment (270 in the experimental group and 220 in the control group). The heterogeneity test showed the heterogeneity to be small (*P* = 0.47, I^2^ = 0), so the solid effect model was used. The meta-analysis revealed that the total effective rate of conventional therapy combined with intravenous vitamin C was higher than that of conventional therapy alone [Z = 5.45, RR = 1.21, 95% CI: (1.13 to 1.30),* P* < 0.00001], as shown in Figure 2.

#### 3.3.2. LDH Level

**Figure 3:**
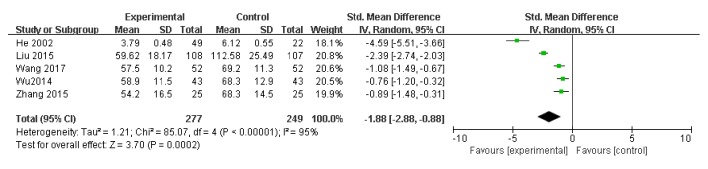
Vitamin C combined with conventional treatment compared with treatment alone in LDH changes after treatment.

Five studies used LDH level as an indicator of the effectiveness of vitamin C-guided interventions. A total of 526 children with myocarditis were included in this assessment (277 in the experimental group and 249 in the control group). The heterogeneity was large (*P* < 0.00001, I^2^ = 0.95); therefore, the random effects model was used. The meta-analysis showed that LDH levels of children with myocarditis who were treated with intravenous vitamin C combined with conventional therapy were lower than those of children with myocarditis who received conventional therapy alone [Z = 3.70, SMD = −1.88, 95% CI (−2.88 to −0.88),* P* = 0.0002], as shown in Figure 3.

#### 3.3.3. CK Level

**Figure 4:**
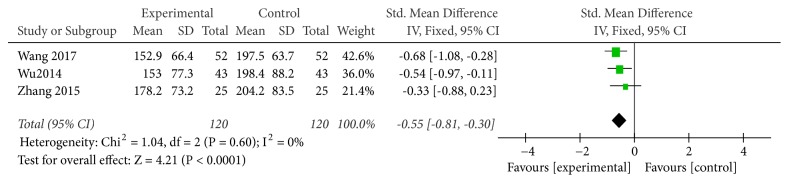
Vitamin C combined with conventional treatment compared with treatment alone in CK changes after treatment.

Five studies used CK level as an indicator of the effectiveness of vitamin C-guided interventions. A total of 240 children with myocarditis were included in this assessment (120 in the experimental group and 120 in the control group). No heterogeneity was observed between the studies (*P* = 0.60, I^2^ = 0%). The solid effect model results showed that after two treatments, the difference in CK levels was significant [Z = 4.21, SMD = −0.55,* P* < 0.0001, 95% CI (−0.81 to −0.30)]. Therefore, intravenous vitamin C combined with conventional therapy is better than conventional therapy alone for reducing CK levels, as shown in Figure 4.

#### 3.3.4. CK-MB Level

**Figure 5:**
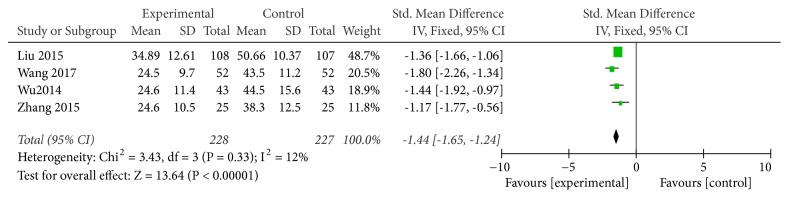
Vitamin C combined with conventional treatment compared with treatment alone in CK-MB changes after treatment.

Four studies used CK-MB levels as an indicator of the effectiveness of vitamin C-guided interventions. A total of 455 children with myocarditis were included in this assessment (228 in the experimental group and 227 in the control group). The heterogeneity was small (*P* = 0.33, I^2^ = 0.12), so the solid effect model was used. The meta-analysis suggested that the CK-MB levels of children with myocarditis who were treated with conventional therapy combined with intravenous vitamin C were lower than those of children with myocarditis who received conventional therapy alone [Z = 13.64, SMD = −1.44, 95% CI (−1.65 to −1.24),* P* < 0.00001], as shown in Figure 5.

#### 3.3.5. CD3 Level

**Figure 6:**
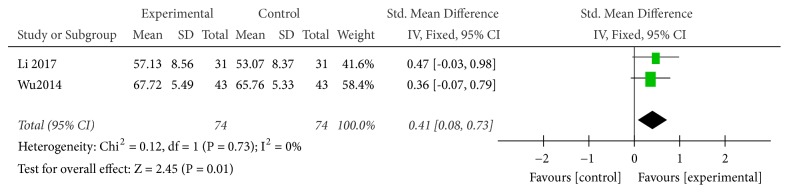
Vitamin C combined with conventional treatment compared with treatment alone in CD3 changes after treatment.

Two studies used CD3 level as an indicator of the effectiveness of vitamin C-guided interventions. A total of 148 children with myocarditis were included in this analysis (74 in the experimental group and 74 in the control group). The heterogeneity was small (*P* = 0.73, I^2^ = 0), so the solid effect model was used. The meta-analysis showed that the CD3 levels of children with myocarditis who were treated with conventional therapy combined with intravenous vitamin C were higher than those of children with myocarditis who received conventional therapy alone [Z = 2.45, SMD = 0.44, 95% CI (0.08 to 0.73),* P* = 0.01], as shown in Figure 6.

#### 3.3.6. CD4 Level

**Figure 7:**
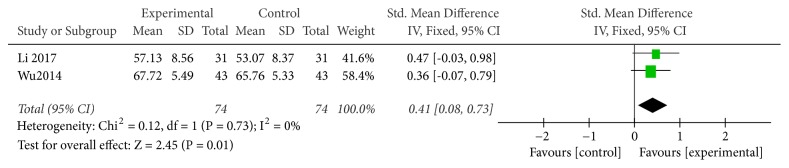
Vitamin C combined with conventional treatment compared with treatment alone in CD4 changes after treatment.

Two studies used CD4 level as an indicator of the effectiveness of vitamin C-guided interventions. A total of 148 children with myocarditis were included in this assessment (74 in the experimental group and 74 in the control group). The heterogeneity was small (*P* = 0.73, I^2^ = 0), so the solid effect model was used. Changes in CD4 levels between the conventional therapy combined with intravenous vitamin C group and the conventional therapy alone group were significant [RR = 0.45, SMD = 0.41, 95% CI (0.08 to 0.73),* P* = 0.01], with the former group exhibiting a higher CD4 content than the latter, as shown in Figure 7.

#### 3.3.7. CD4/CD8 Ratio

**Figure 8:**
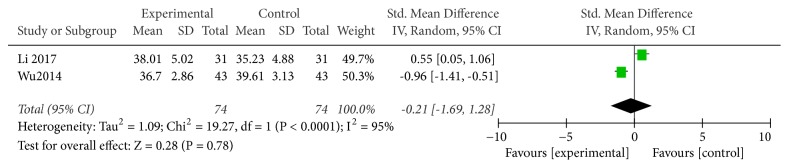
Vitamin C combined with conventional treatment compared with treatment alone in CD4CD8 changes after treatment.

Two studies used CD4/CD8 ratio as an indicator of the effectiveness of vitamin C-guided interventions. A total of 148 children with myocarditis were involved in this assessment (74 in the experimental group and 74 in the control group). The heterogeneity was large (*P* < 0.00001, I^2^ = 0.95), so the random effects model was used. There was no significant difference in changes in CD4/CD8 ratios between the conventional therapy combined with intravenous vitamin C group and the conventional therapy alone group [RR = −0.21, SMD = −0.21, 95% CI (−1.69 to 1.28),* P* = 0.78], as shown in Figure 8.

#### 3.3.8. Adverse Reactions

Of the eight studies included, one reported no significant adverse reaction, and the remaining seven did not mention adverse reactions.

#### 3.3.9. Publication Bias

**Figure 9:**
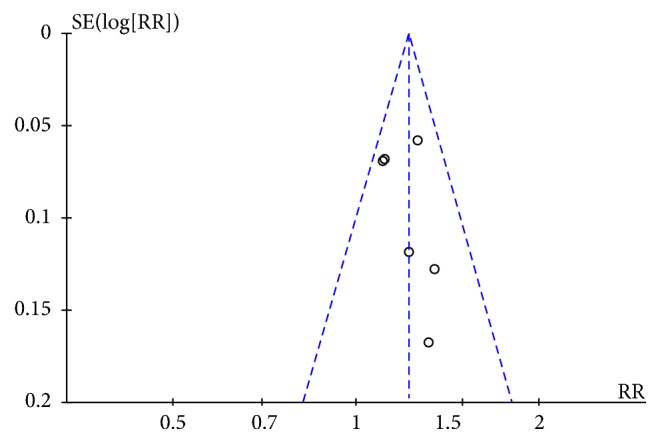
Funnel plot.

As summarized in Figure 9, a funnel plot was applied to evaluate the publication bias of all 12 studies. The outcomes suggested that there was little publication bias.

## 4. Discussion

### 4.1. Analysis of Vitamin C Efficacy

The meta-analysis explained that the total effective rate in children with myocarditis who were treated with conventional therapy combined with intravenous vitamin C was higher than that in children with myocarditis who received conventional therapy alone [RR = 1.21, 95% CI: (1.13 to 1.30)]; i.e., the total effective rate of conventional therapy combined with intravenous vitamin C for children with myocarditis can be increased by approximately 20% compared with that of conventional therapy alone. LDH, CK, and CK-MB levels were the parameters used to evaluate myocardial enzyme levels. This meta-analysis reported that LDH, CK, and CK-MB levels were lower in the intravenous vitamin C combined with conventional therapy group than in the conventional therapy alone group. The results were as follows: LDH: SMD = −1.88, 95% CI (−2.88 to −0.88); CK: SMD = −0.55, 95% CI (−0.81 to −0.30); and CK-MB: SMD = −1.44, 95% CI (−1.65 to −1.24). Intravenous vitamin C combined with conventional therapy can reduce myocardial enzyme levels more effectively than conventional therapy. CD3 and CD4 are T lymphocyte subsets; the meta-analysis revealed that both CD3 and CD4 levels were higher after intravenous vitamin C combined with conventional therapy than after conventional therapy alone (SMD = 0.44, 95% CI (0.08–0.73) and SMD = 0.41, 95% CI (0.08–0.73), respectively). There was no significant difference between the two groups regarding changes in CD4/CD8 ratios (*P* = 0.78). According to the meta-analysis results of the above indicators, intravenous vitamin C on the basis of conventional therapy in children with viral myocarditis may effectively improve clinical efficacy.

### 4.2. Limitations

There are several limitations to the present study. First, this study failed to evaluate other indicators that can completely reflect heart conditions. Second, the studies included in this meta-analysis had small sample sizes, and the sample sizes differed significantly across studies. Some of the literature data varied substantially, and some factors affecting the results were not mentioned. Multicenter and large-scale randomized trials are relatively rare. In addition, the included studies had limited descriptions of randomization methods, allocation schemes, and blinding methods; therefore, there may be selection and measurement biases. Considering the restriction of including only Chinese and English language studies, it is impossible to exclude the possibility of language bias. Because of the limitations of the quantity and quality of the included studies, the results of this meta-analysis may be biased, and further high-quality experimental research is warranted. The mechanism through which vitamin C exerts a positive effect in the treatment of viral myocarditis in children requires further research.

### 4.3. Application Prospects

Myocardial zymogram changes have been regarded as one of the diagnostic indicators of viral myocarditis and have important clinical significance. Among them, CK and CK-MB levels have the greatest significance. The positive rate of CK-MB increase in acute myocarditis varies from 20% to 70%, which may be related to the detection time. CK-MM is almost entirely present in the serum of normal individuals, accounting for 94%–96%, whereas CK-MB is <5%. If serum CK-MB level is significantly elevated, it indicates that the myocardium is evidently involved. It is typically believed that the serum CK-MB level is a specific index of myocardial injury when it is >6% of the total activity [17]. CK, in humans, shows a rapid increase in cases of myocardial ischemia or subendocardial infarction, and it is an important indicator for clinical diagnosis of myocarditis. The myocardial enzyme spectrum in children is typically 2–3 times higher than that in adults. When LDH level increases, it exhibits tissue specificity in human body parts, and it is noteworthy that its increase corresponds to the occurrence of myocardial lesions [14]. It is currently believed that the pathogenesis of viral myocarditis is closely related to immune function [18]. T lymphocytes are the effector cells of cellular immunity as well as important immunoregulatory cells, in addition to being a heterogeneous multifunctional group. CD3+ represents the total number of mature T cells in the peripheral blood, and the increase of CD3+ indicates the enhancement of T cell immune function; CD4+ is a T cell subset with a helper–inducer function. The increase of CD4+ indicates the increase of immunoglobulin produced by B cells and the improvement of cellular immunity. CD8+ is an inhibitory cell, and the increase of CD8+ indicates immunosuppression. The CD4+/CD8+ ratio reflects the equilibrium between induction and inhibition and is the most important indicator of environmental stability in the human immune system [19]. T cells are immune cells that are functional in eliminating pathogenic bacteria and increasing the number of T cells. They may effectively enhance the cellular immune function of children, inhibit the reproduction of pathogenic bacteria in vivo, reduce the damage of pathogenic bacteria to the human body, decrease the number of myocardial enzymes in children, and effectively stabilize the growth environment of myocardial cells [1]. The meta-analyses conducted in the present study shows that conventional therapy combined with intravenous vitamin C could substantially reduce LDH, CK, and CK-MB levels and increase CD3 and CD4 compared with conventional therapy.

Vitamin C is a free radical scavenger that can inhibit the production of oxygen free radicals [20], prevent inflammatory cells from releasing superoxide free radicals, and reduce myocardial damage. Moreover, it can enhance the body's resistance to infection, increase coronary blood flow [21], and improve myocardial metabolism to help repair myocarditis damage, thereby greatly reducing the course of disease and helping children recover and improve their disposition [22]. It is widely believed that the use of vitamin C has multiple benefits. For example, an appropriate amount of vitamin C can help resolve facial skin issues, prevent cardiovascular diseases, improve immunity, relieve fatigue, and help treat cancer and tumors [23–28]. However, there are reports of adverse reactions, such as oxalate nephropathy, renal failure, phlebitis, and iron excess [29–32]. The meta-analysis results suggest that conventional therapy combined with vitamin C is effective in the treatment of viral myocarditis in children, and the results have certain clinical significance. If children with viral myocarditis are routinely treated and vitamins are used, the course of treatment can be effectively shortened. Vitamin C is widely available and inexpensive and has few adverse effects, making it worth promoting.

## 5. Conclusion

Vitamin C combined with conventional therapy for the treatment of viral myocarditis in children displays a better total effective rate and results in lower LDH, CK, and CK-MB levels than simple, conventional therapy.

## Figures and Tables

**Table 1:** Characteristics of included studies.

author	time	Proportion	experiment		control		Evaluation indicator
		male/female	cases	Vitamin C	cases	Vitamin C	
Chenshiping [10]	2006	39/23	32	100~200mg/Kg+RT	30	RT	total effective rate
Hezenghong [11]	2002	109/55	112	200mg/Kg+RT	52	RT	LDH, AST
Huzhihong [12]	2005	24/22	23	100mg/Kg+RT	23	RT	total effective rate
Liguangchao [13]	2007	unclear	31	175mg/Kg+RT	31	RT	total effective rate, CD3, CD4, CD4/CD8
Liuying [14]	2015	110/105	108	150~200mg/Kg+RT	107	RT	total effective rate, LDH, CK-MB
Wangmeng [15]	2017	62/42	52	250mg/Kg+RT	52	RT	total effective rate, LDH, CK, CK-MB,
Wukeyi [16]	2014	47/39	43	150~200mg/Kg+RT	43	RT	total effective rate, LDH, CK, CK-MB, CTNI, CD3, CD4, CD4/CD8
Zhangzhen [17]	2015	27/23	25	150~200mg/Kg+RT	25	RT	LDH,CK, CK-MB

**Table 2:** Quality evaluation of the included studies.

Included studies	Random allocation	Allocation concealment	Double blind method	Evaluation of blindness	Data integrity	Selective report	others
Chen 2006	Unknown	No	No	No	Yes	Yes	Yes
He 2002	No	No	No	No	Yes	Yes	Yes
Hu 2005	Unknown	No	No	No	Yes	Yes	Yes
Li 2007	Yes	No	No	No	Yes	Yes	Yes
Liu 2015	Unknown	No	No	No	Yes	Yes	Yes
Wang 2017	Yes	No	No	No	Yes	Yes	Yes
Wu 2014	Yes	No	No	No	Yes	Yes	Yes
Zhang 2015	Unknown	No	No	No	Yes	Yes	Yes

## Data Availability

The data used to support the findings of this study are included within the article.
